# Maria M. Mota: Bringing *Plasmodium* Liver Infection to the Centre Stage of Malaria Research

**DOI:** 10.3389/fcimb.2022.851484

**Published:** 2022-02-08

**Authors:** Sílvia Portugal, Ana Rodriguez, Miguel Prudêncio

**Affiliations:** ^1^ Max Planck Institute for Infection Biology, Berlin, Germany; ^2^ Department of Microbiology, New York University School of Medicine, New York City, NY, United States; ^3^ Instituto de Medicina Molecular, Faculdade de Medicina, Universidade de Lisboa, Lisboa, Portugal

**Keywords:** malaria, liver stage, sporozoites, *Plasmodium*, hepatocyte

## Introduction

Mammalian infection by malaria parasites is initiated by the injection of *Plasmodium* sporozoites into the host’s skin in preparation for a blood meal by an infected female *Anopheles* mosquito. Sporozoites then travel to the liver, where they invade hepatocytes and initiate an asymptomatic phase of asexual replication, known as the liver stage of infection. This process culminates in the release of thousands of newly-formed red blood cell-infective merozoites, which are responsible for malaria-associated pathology (Prudencio et al., 2006). Although the liver stage of the *Plasmodium* life cycle constitutes the initial and obligatory step of mammalian infection by malaria parasites, it remained as a biological black box for decades since its discovery by Shortt, Garnham and their team in 1948 (Shortt et al., 1948). Its clinically silent nature, coupled with the inherent limitations of its experimental address, meant that liver infection by *Plasmodium* remained largely unknown by researchers for decades. However, it is now known that this pivotal phase of the malaria parasite’s life cycle plays critical roles in the establishment of mammalian infection by *Plasmodium*, in the host’s response to the parasite, and in the outcome of disease. The *Plasmodium*-host hepatic interface is now commonly regarded not only as an ideal target for malaria prophylaxis and vaccination, but also as the site where a unique and extremely rich array of molecular interactions take place. And few people have contributed as much as Maria M. Mota to unveiling key features of the liver stage of the malaria parasite’s life cycle. By engaging the most advanced *in vitro* and rodent models of *Plasmodium* infection (Langhorne et al., 2011; Prudencio et al., 2011; Zuzarte-Luis et al., 2014), Maria decisively helped to bring this once-relatively obscure phase of the parasite’s life cycle to the limelight of malaria research.

Maria Manuel Mota obtained her BSc in Biology and her MSc in Immunology in 1992 and 1994, respectively, at the University of Porto, Portugal, followed by a PhD in Molecular Parasitology by the University College London in 1998. She then carried out her post-doctoral studies on host-parasite interactions in Victor Nussenzweig’s lab at the New York University Medical School from 1999 to 2002. On this year, she returned to her home country of Portugal to become a group leader, initially at Instituto Gulbenkian de Ciência (IGC), and subsequently at Instituto de Medicina Molecular (iMM), where she became the Executive Director in 2014.

The authors of this article have been fortunate enough to interact closely with Maria M. Mota at different stages of her career. Maria spent part of her time at the laboratory of AR during her postdoctoral studies at the New York University; MP joined Maria’s group as a postdoctoral researcher soon after she became an independent scientist, and was a staff scientist in her laboratory before becoming an independent group leader at Lisbon’s iMM; and SP was Maria’s PhD student at a time when her position as a key player in malaria research was well solidified and internationally recognized. Each one of us has had the unique opportunity to perceive Maria’s unique scientific drive, her contagious enthusiasm, and her unabashed commitment to research. We are all indebted to Maria for her continued support of our own scientific careers but, above all, we feel very fortunate to have witnessed some of the most remarkable discoveries made in the field of liver stage *Plasmodium* infection. As Maria’s career as an independent scientist reaches its 20^th^ anniversary, this is our account of some of her most relevant scientific achievements of the last two decades.

## Re-Designing the Life Cycle of Malaria Parasites

In 2001, Maria M. Mota, working in the laboratory of AR, demonstrated for the first time that *Plasmodium* sporozoites traverse several hepatocytes before productively invading a final cell (Mota et al., 2001), and suggested that this migration might activate sporozoites for productive invasion of hepatocytes (Mota et al., 2002). Maria’s work subsequently showed that hepatocyte growth factor (HGF), a molecule released by host cells upon wounding by traversing sporozoites, interacts with its receptor MET to promote the parasites’ intrahepatic development (Carrolo et al., 2003) and to protect *Plasmodium*-infected cells from apoptosis (Leiriao et al., 2005). While the exact reasons for, and the implications of the parasite’s migratory behavior remain incompletely elucidated, in the words of Victor Nussenzweig, Maria M. Mota’s supervisor and mentor at the New York University, “Maria’s finding literally changed the textbook representation of the life cycle of *Plasmodium* parasites”.

## Host Genes and Drug Targets

As Maria initiated her independent research career in Portugal, her focus on the study of the liver stage of *Plasmodium* infection intensified and expanded, and she soon reported the first transcriptome profile of the hepatic host cell throughout infection by malaria parasites (Albuquerque et al., 2009). Soon thereafter, Maria’s lab pioneered the use of RNA interference (RNAi) to identify host factors at play during hepatic *Plasmodium* infection. An RNAi screen of the entire human kinome implicated five host kinases in this process, further establishing a role for PKCς on hepatocyte invasion by sporozoites (Prudencio et al., 2008). A parallel RNAi screen additionally unveiled the hepatic host’s scavenger receptor SR-BI as playing a critical role in both the sporozoite’s ability to invade liver cells and to develop intracellularly (Rodrigues et al., 2008). During this period, the work of Maria’s team also led to the identification of genistein as a potential drug for malaria prophylaxis (Cunha-Rodrigues et al., 2008), to the demonstration that *Plasmodium* liver infection can be inhibited by a small molecule inhibitor of signal peptide peptidase, with an impact on malaria severity (Parvanova et al., 2009), and that CpG phosphothioate oligodeoxynucleotides can act directly on *Plasmodium* sporozoites to inhibit their gliding motility, cell traversal ability and capacity to invade hepatic cells (Liehl et al., 2010). In 2012, she was deeply involved in pioneering drug screens targeting the liver stage of the parasite’s life cycle, which identified decoquinate as a potent multi-stage antiplasmodial drug (da Cruz et al., 2012), and revealed that hepatic malaria parasites are vulnerable to diverse chemical scaffolds (Derbyshire et al., 2012). One year later, her lab identified torins as fast-acting anti-plasmodial compounds that efficiently target the parasite’s liver and blood stages in a manner that is independent of those drug’s canonical target, the mammalian target of rapamycin (mTOR) kinase (Hanson et al., 2013). Collectively, these and other achievements helped to define Maria’s position at the forefront of liver stage *Plasmodium* infection research, a status that never ceased to solidify.

## The Pathogenesis of Severe Malaria

Always acutely aware of the pathology that ensues during the erythrocytic phase of the malaria parasite’s life cycle, Maria’s lab helped unveil heme oxygenase-1 (HO-1) as a host factor that not only promotes liver infection by *Plasmodium* parasites (Epiphanio et al., 2008), but that also plays a crucial role in the development of experimental cerebral malaria (Pamplona et al., 2007). A few years later, the team established a DBA/2 mouse model for the study of *Plasmodium*-induced experimental acute lung injury, showing that this life-threatening condition is promoted by the host’s vascular endothelial growth factor (VEGF), and can be inhibited by carbon monoxide’s anti-inflammatory action (Epiphanio et al., 2010). These achievements furthered our knowledge of malaria pathogenesis, and remain as a testimony of Maria’s ability to think beyond the liver stage of infection and seeking solutions to combat the most grievous forms of malaria disease.

## Hepatocyte Invasion by *Plasmodium* Sporozoites

One of the questions that had puzzled the community for decades was how *Plasmodium* sporozoites could engage into a phase of intra-hepatic differentiation and multiplication so promptly after transitioning from the mosquito salivary glands to hepatocytes. Maria’s lab contributed pivotally to our current knowledge of the molecular determinants of this process, by unveiling a Pumilio-2 (Puf2)-dependent post-translational repression mechanism that controls that transition (Gomes-Santos et al., 2011). Very recently, her team challenged the dogma that hepatocytes are passive players during the process of their invasion by sporozoites. This work showed that the pore-forming activity of the parasite’s exported protein 2 (EXP2) triggers a response from the hepatic host cell, which leads to the production of acid sphingomyelinase. This enzyme then plays a critical role in the repair of the host cell membrane, which is key for sporozoite invasion and establishment in hepatocytes (Mello-Vieira et al., 2020). This finding is a perfect illustration of Maria’s ability to push the boundaries of knowledge beyond the *status quo*, by unveiling an active role for the hepatic host cell in a process long thought to depend solely on the parasite.

## New Tools to Study Liver Infection by *Plasmodium*

Throughout her career, Maria contributed several new instruments to the fight against malaria, thorough the establishment of new tools and platforms for the investigation of *Plasmodium* liver infection. Among other achievements, she and her team demonstrated the usefulness of transgenic malaria parasites expressing fluorescent or luminescent reporter genes as invaluable tools to identify and quantify *Plasmodium* infection, not only in the liver (Prudencio et al., 2008; Ploemen et al., 2009), but also in the blood (Zuzarte-Luis et al., 2014). During a sabbatical in Sangeeta Bhatia's laboratory at the Massachusetts Institute of Technology (MIT), Maria contributed to the generation of a human liver *in vitro* platform that is able to sustain the development of human malaria parasites (March et al., 2013), and showed that human induced pluripotent stem cell-derived hepatocyte-like cells support infection by multiple *Plasmodium* sporozoite species (Ng et al., 2015). These achievements, alongside the more recent establishment of a humanized mouse model bearing an ectopic artificial liver that can be infected by human malaria parasites (Ng et al., 2017), provided the community with invaluable new tools for the investigation of *Plasmodium* liver infection and the assessment of anti-plasmodial compounds.

## Immune Responses to *Plasmodium* Liver Infection

The asymptomatic nature of the liver stage of *Plasmodium* infection contributed to the long-held assumption that this phase of the parasite’s life cycle was also immunologically silent. Maria’s work effectively challenged this view and showed that, quite the contrary, this a phase when a rich array of immune responses take place, with a clear impact on infection and pathology. Her early work on HO-1 had already shown that liver infection elicits a potent inflammatory response in the host (Epiphanio et al., 2008). A few years later, work from Maria’s lab showed that *Plasmodium* liver stages induce a potent type I interferon (IFN) response that prompts host cell sensors to activate an immune cell-mediated response (Liehl et al., 2014), the magnitude of which effectively inhibit re-infection in an IFN- γ -dependent way (Liehl et al., 2015). On the other hand, the lab also showed that IFN-γ produced by γδ-T cells upon liver-stage infection can have a deleterious effect on the host and promote experimental cerebral malaria (ECM) pathogenesis (Ribot et al., 2019). The enormous potential of the liver stage of infection for immunization against malaria is now firmly established, and Maria has made important contributions to the whole-sporozoite-based vaccination approaches. In 2007, her team showed that P36p-deficient *P. berghei* sporozoites can induce protection against a subsequent challenge with fully infectious parasites (Douradinha et al., 2007), contributing to the exploitation of genetically-attenuated parasites (GAP) as vaccination agents against malaria. More than 10 years later, she was involved in a pioneering study by MP that established the proof-of-concept of employing genetically modified rodent malaria parasites, expressing antigens of their human-infective counterparts, as a platform for vaccination against human malaria (Mendes et al., 2018). Collectively, these achievements helped redefine the liver stage of *Plasmodium* infection as an immunologically rich phase of the parasite’s life cycle, once again challenging the prevailing dogma of its immune passiveness.

## Cross-Talk Between Mammalian Infection Stages

One of Maria’s characteristics is her view of *Plasmodium* as a complex organism, whose different life cycle stages in the mammalian host should not be viewed independently of each other, as a bidirectional cross-talk exists, in which one stage of mammalian infection may impact the establishment and progression of the other. The identification of HO-1 as a host factor that operates both during the liver (Epiphanio et al., 2008) and the blood (Pamplona et al., 2007) stages of infection by Maria’s team nicely illustrates her holistic view of mammalian infection by *Plasmodium*. This is perhaps even better exemplified by Maria’s investigation of the direct impact of blood stage *Plasmodium* on a subsequent hepatic infection by the same parasite. This work, carried out during SP’s PhD, revealed for the first time that an ongoing *Plasmodium* blood stage infection potently inhibits a subsequent infection by *Plasmodium* sporozoites. An in depth look at the mechanisms behind this inhibition showed that this process depends at least partly on the iron regulatory hormone hepcidin. This finding unveiled a host-mediated quorum-sensing-like mechanism that regulates superinfection by malaria parasites in regions of high disease endemicity (Portugal et al., 2011).

## Exploitation of Host Resources by *Plasmodium* and Host Responses to Infection

Among the most pervasive questions throughout Maria M. Mota’s scientific career are “how does the parasite exploit the host for its own benefit?” and “how does the host respond to the invading parasite?” Maria’s curiosity about the role of host cell factors and host-mediated processes during liver infection by *Plasmodium* prompted an array of major achievements that significantly furthered our understanding of the biology of the malaria parasite’s sporozoite and liver stages. Looking closely into the infected liver cell, Maria’s lab described for the first time a highly dynamic set of hepatocyte actin reorganization events that occur around developing *Plasmodium* parasites inside hepatic cells, which may contribute to their elimination during development in the liver (Gomes-Santos et al., 2012). Soon afterwards, the lab demonstrated that hepatic *Plasmodium* parasites take up phosphatidylcholine from the host to support their replication (Itoe et al., 2014), and that *Plasmodium*’s vacuolar iron-transporter (VIT) homologue plays an important role in iron detoxification, contributing to the parasite’s normal development in the liver (Slavic et al., 2016). Maria’s team further showed that liver infection by malaria parasites is facilitated by the host’s hepatic endoplasmic reticulum (ER)-resident unfolded protein response (UPR) (Inacio et al., 2015), whereas signaling by the host’s AMP-activated protein kinase (AMPK) exerts a suppressive effect on hepatic infection (Ruivo et al., 2016). Having contributed to the demonstration that the host’s autophagy machinery contributes to the *Plasmodium*’s hepatic development (Thieleke-Matos et al., 2016), Maria’s lab later showed that *Plasmodium* relies on its own upregulated in sporozoites 3 (UIS3) protein to outcompete the autophagy marker microtubule-associated protein 1 light chain 3 (LC3), decreasing its binding to the parasitophorous vacuole membrane (PVM), and thereby avoiding elimination (Real et al., 2018). Maria and her team then showed that this interaction between the parasite’s UIS3 and the host’s LC3 molecules can be chemically disrupted, inhibiting the parasite’s ability to evade the host autophagy response and leading to its elimination (Setua et al., 2020). This array of discoveries has brought us closer to understanding why *Plasmodium* sporozoites “choose” the hepatocyte as their initial site of replication in the mammalian host, a key question in this field of research.

## Host-Mediated Modulation of Infection

Maria’s conviction that *Plasmodium* infection can be modulated by the host prompted her and her team to investigate whether the host’s nutritional status influences the course of infection and pathology. This study unveiled a novel mechanism whereby the *Plasmodium* kinase KIN acts as a sensor that enables the parasite to modulate its replication rate in accordance with the host’s nutritional status (Mancio-Silva et al., 2017). This discovery was reported close to another one, addressing the modulation of *Plasmodium* liver infection by the host’s dietary intake. This study showed that the oxidative stress induced by a high-fat diet causes the death of intra-hepatic parasites, leading to a major decrease in the overall *Plasmodium* liver load, and significantly decreasing the severity of the ensuing disease (Zuzarte-Luis et al., 2017). These discoveries have contributed decisively to enhancing the notion that the mammalian host plays a critical role in the development of malaria disease, and that the latter can be influenced through the modulation of the parasite’s environment inside the former.

## Final Remarks

Maria’s achievements as a malaria researcher have earned her worldwide recognition in the community. Her appointments as a Howard Hughes Medical Institute (HHMI) International Research Scholar from 2005 to 2010, and as a European Molecular Biology Organization (EMBO) Member in 2016 constitute significant recognitions of both her accomplishments and her potential. Besides her scientific excellence, Maria has a captivating personality and is an engaging communicator, who has brought malaria research to the spotlight in Portugal and beyond, through her multiple public appearances in the media and her ability to entice her audiences. A staunch advocate for women’s rights and equal education opportunities, she commonly features in the list of the most influential women in Portugal and has earned numerous national and international awards that further enhanced her standing in the malaria research community. She has helped foster iMM’s reputation and recognition at both the national and international levels, and her actions have had an enormous impact on science policies in Portugal as a whole. She has inspired and guided more than one generation of scientists, many of whom have grown to lead their independent research labs.

Maria M. Mota’s scientific career is an inspiring example of commitment to science, enthusiasm and excellence. She was able to carve a research niche that earned her international reputation and contributed greatly to Portugal’s prominence in malaria research over the last couple of decades (Prudencio, 2021). Naturally, her achievements owe immensely to the efforts and dedication of her research team. Over the years, her laboratory has hosted numerous postdoctoral researchers, PhD and Master’s students, laboratory technicians and managers, and visiting scientists, all of whom have been inspired by Maria, and all of whom have contributed enormously to the success of her research. Equally relevant, the respect earned by Maria in the scientific community has enabled her to establish an extended network of outstanding collaborators, to whom she also owes much of her success. As Maria likes to say, ideas appear when people come together and talk to each other about their science. Maria’s ability to foster discussion, both within and beyond her own lab, has always been one of the distinctive features of her scientific drive. As Executive Director of iMM, Maria created the institute’s motto “chasing questions”, because she deeply believes that the formulation of the most creative questions is the key to all major scientific advances.

Maria’s brain is constantly teeming with new interrogations that drive her creativity and fuel her passion for science. While it may not be easy to predict exactly what scientific achievements Maria’s future will bring, it is certainly not hard to forecast that she will continue challenging the borders of our knowledge of the liver stage of *Plasmodium* infection. As such, we can be pretty confident that many fascinating discoveries still lie ahead in her path through science. Stay tuned!

## Author Contributions

All authors contributed to the article and approved the submitted version.

## Conflict of Interest

The authors declare that the research was conducted in the absence of any commercial or financial relationships that could be construed as a potential conflict of interest.

## Publisher’s Note

All claims expressed in this article are solely those of the authors and do not necessarily represent those of their affiliated organizations, or those of the publisher, the editors and the reviewers. Any product that may be evaluated in this article, or claim that may be made by its manufacturer, is not guaranteed or endorsed by the publisher.
